# Evaluation of the dosimetric properties of a diode detector for small field proton radiosurgery

**DOI:** 10.1120/jacmp.v16i6.5391

**Published:** 2015-11-08

**Authors:** Grant A. McAuley, Anthony V. Teran, Jerry D. Slater, James M. Slater, Andrew J. Wroe

**Affiliations:** ^1^ Department of Radiation Medicine Loma Linda University Loma Linda CA; ^2^ Department of Radiation Medicine Loma Linda University Medical Center Loma Linda CA USA

**Keywords:** proton therapy, radiosurgery, small field dosimetry, silicon diode radiation detectors

## Abstract

The small fields and sharp gradients typically encountered in proton radiosurgery require high spatial resolution dosimetric measurements, especially below 1–2 cm diameters. Radiochromic film provides high resolution, but requires postprocessing and special handling. Promising alternatives are diode detectors with small sensitive volumes (SV) that are capable of high resolution and real‐time dose acquisition. In this study we evaluated the PTW PR60020 proton dosimetry diode using radiation fields and beam energies relevant to radiosurgery applications. Energies of 127 and 157 MeV (9.7 to 15 cm range) and initial diameters of 8, 10, 12, and 20 mm were delivered using single‐stage scattering and four modulations (0, 15, 30, and 60 mm) to a water tank in our treatment room. Depth dose and beam profile data were compared with PTW Markus N23343 ionization chamber, EBT2 Gafchromic film, and Monte Carlo simulations. Transverse dose profiles were measured using the diode in "edge‐on" orientation or EBT2 film. Diode response was linear with respect to dose, uniform with dose rate, and showed an orientation‐dependent (i.e., beam parallel to, or perpendicular to, detector axis) response of less than 1%. Diode vs. Markus depth‐dose profiles, as well as Markus relative dose ratio vs. simulated dose‐weighted average lineal energy plots, suggest that any LET‐dependent diode response is negligible from particle entrance up to the very distal portion of the SOBP for the energies tested. Finally, while not possible with the ionization chamber due to partial volume effects, accurate diode depth‐dose measurements of 8, 10, and 12 mm diameter beams were obtained compared to Monte Carlo simulations. Because of the small SV that allows measurements without partial volume effects and the capability of submillimeter resolution (in edge‐on orientation) that is crucial for small fields and high‐dose gradients (e.g., penumbra, distal edge), as well as negligible LET dependence over nearly the full the SOBP, the PTW proton diode proved to be a useful high‐resolution, real‐time metrology device for small proton field radiation measurements such as would be encountered in radiosurgery applications.

PACS numbers: 87.56.‐v, 87.56.jf, 87.56.Fc

## I. INTRODUCTION

With improvements in imaging, patient alignment, and beam delivery technology, there is an emerging trend in radiation therapy to treat smaller lesions with higher doses in fewer fractions. These techniques are not limited to photon therapy modalities, but are being used increasingly in proton therapy, and require accurate and high‐resolution dosimetry in order to achieve optimal effectiveness and maintain patient safety. Additionally, in proton therapy we are seeing an emergence of active or pencil beam proton scanning, where small proton beams are magnetically scanned to deliver a conformal dose to complex targets. These pencil beams require high‐resolution dosimetry for both beam commissioning and treatment planning system input, as well as for routine QA.

Small proton therapy fields, such as those typically encountered in proton radiosurgery, SBRT, and pencil beam scanning applications, require high‐resolution dosimetric measurements for both point dose measurements and beam profile analysis. However, as field sizes are reduced below 1–2 cm in diameter, dosimetry becomes increasingly difficult as the spatial resolution limits of many standard metrology apparatus are approached. Furthermore, protons have the added complication of varying LET as a function of depth (or energy) that can strongly impact detector response. Radiochromic film provides high‐resolution measurements, but exhibit a LET dependent response, requires postprocessing, special handling, and supporting data acquisitions for calibration and to control for artifacts.^1^, ^2^, ^3^, ^4^, ^5^ A promising alternative is provided by diode detectors with small rugged sensitive volumes (SV) that are capable of high resolution and real‐time dose acquisition, and that are now commercially available.

While several studies characterizing diode detectors used for proton dose measurements have been published over the last four decades, these studies have often shown mixed or less‐than‐desirable results when compared to ionization chamber detectors.^6^, ^7^, ^8^, ^9^, ^10^, ^11^, ^12^, ^13^, ^14^, ^15^, ^16^, ^17^ However, Grusell and Medin^(11)^ showed that a highly doped p‐type Si detector designed for use with protons had a response consistent with ionization chambers, which are considered the gold standard for dose measurements. In the present study, we present results from an evaluation of the commercially available PTW PR60020 p‐type diode detector specifically indicated by the manufacturer for proton dose measurements. To assess performance with respect to spatial resolution and LET dependence, the diode's response was compared to Gafchromic EBT2 film, a PTW Markus N23343 parallel plate ionization chamber, and Geant4‐based Monte Carlo simulations. Both depth‐dose and lateral profiles of beams typically used in radiosurgical applications were evaluated.

## II. MATERIALS AND METHODS

Data was collected in the Gantry 1 treatment room of the James M. Slater MD Proton Therapy and Research Center at the Loma Linda University Medical Center (LLUMC).^(18)^ Comparisons of diode data with ion chamber or film were conducted using our standard nominal radiosurgery energies of 127 MeV and 157 MeV through a single‐stage scattering system, corresponding to a range of 9.7 and 15 cm in water, respectively. The single‐stage scattering system provides a maximum field size of 4 cm diameter, with collimation provided via a dedicated SRS cone that attaches to the end of the proton nozzle. This system allows for minimal air‐gap between collimator and patient, ensuring that the penumbra is minimized while maintaining a high dose rate. An array of beam modulations (15 mm (Mod15), 30 mm (Mod30), and 60 mm (Mod60)) were tested that are typical for radiosurgical applications, including unmodulated cases, which can be used for functional radiosurgery. The unmodulated beams are also representative of pencil beams used in active beam scanning delivery. To avoid partial volume effects with the larger volume ion chamber, the beam diameter was 20 mm for all experiments, except where depth‐dose data were acquired using 127 MeV 8, 10, and 12 mm beams, to test how accurately the diode can record dose from beams with diameters of the order of 1 cm. Analogous Monte Carlo simulations were performed for all energies, modulation, and beam diameters for comparison. Central axis depth‐dose profiles and cross‐profiles at various water‐equivalent depths (WED) were compared. The details of these measurement parameters are summarized in Table 1.

**Table 1:** Proton beam properties for both measured and simulated data

*Energy (MeV)*	*Modulation (mm)*	*Diameter (mm)*	*Cross Profile WED (mm)*
127	None	20	N/A
127	None	8	N/A
127	None	10	N/A
127	None	12	N/A
127	15	20	33.0 & 89.6
127	30	20	33.0 & 82.0
157	None	20	N/A
157	30	20	33.0 & 135.0
157	60	20	33.0 & 120.0

### A. Diode and ion chamber metrology

**Figure 1:**
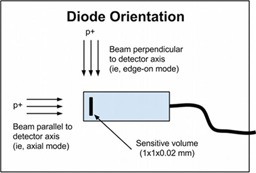
Diode orientation: axial orientation = beam parallel to detector axis; edge‐on orientation = beam perpendicular to axis.

The PTW PR60020 (PTW‐Freiburg, Germany) is a p‐type silicon diode detector that has physical dimensions of 7 mm diameter and 45.5 mm length. This diode has a single cylindrical sensitive volume (SV) with a cross‐sectional area of 1 mm^2^ and a thickness of 20 μm, with a water‐equivalent window thickness of 1.33 mm. Precursory experiments were performed to evaluate the diode response as a function of dose, dose rate, and diode orientation. To test linearity of the diode response with respect to dose, the diode was placed at a 42 mm WED in a water tank and nine different doses, ranging from 0.6 to 23 Gy, were delivered to the detector, and accumulated charge was plotted against dose. To assess for uniformity of diode response with respect to dose rate, beam was delivered to the detector at 14 different dose rates ranging from 0.7 to 2.3 cGy/s and normalized dose per MU was plotted against dose rate. To test for diode damage, the baseline detector response was determined by averaging its response from three 2±2% Gy irradiations. This measurement was completed three more times but with 56±0.5% Gy delivered before each set of measurements. The average response from each measurement set was plotted versus accumulated dose normalized with respect to the initial baseline value. Finally, data were collected with the diode in two different orientations: the diode was positioned such that the beam was parallel to the detector axis (axial orientation), or perpendicular to the axis (edge‐on orientation)^10^, ^19^ (see Fig. 1), with the SV centered at 42 mm WED for each orientation for comparison. Four replicates of 170±3.5% cGy were delivered in each orientation, and the ratio of charge to MU was normalized to the maximum ratio of all eight data points.

**Figure 2:**
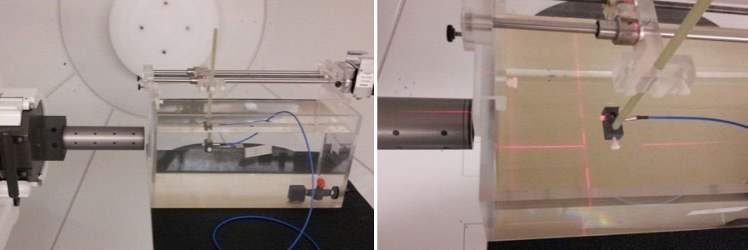
Experimental setup for depth dose measurements using the diode detector in axial orientation.

Depth‐dose profiles were measured in a water tank using both the PR60020 diode and a PTW Markus N23343 plane‐parallel ion chamber with beam incident axial to each detector (Fig. 2). The PTW Markus N23343 is a plane parallel ionization chamber with physical dimensions of 30 mm diameter and 14 mm length. It has a SV of 0.055 cm^3^ vented to air with a 5.3 mm diameter collector, 0.2 mm guard ring width, and a 0.03 mm polyethylene entrance window with a water‐equivalent window thickness of 1.06 mm. Due to its high in‐plane spatial resolution, the Markus ionization chamber is the standard for depth‐dose measurements at our institution for field sizes greater than 1.5 cm diameter. For fields less than 1.5 cm diameter, depth‐dose profiles generated by a validated Monte Carlo simulation would be used for comparison with the PR60020 diode under investigation here.

**Figure 3:**
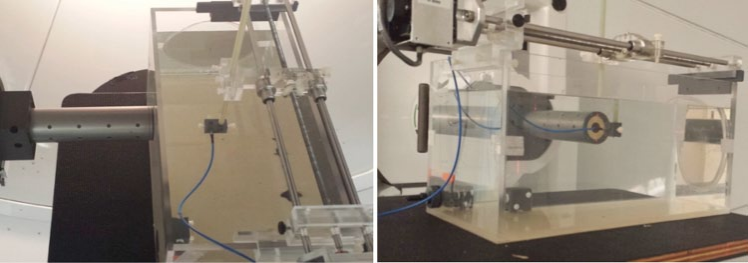
Experimental setup for transverse profile measurements using the diode detector in "edge on" orientation.

The water tank used in this study is equipped with a probe positioning track consisting of a linear corkscrew rail and stepper motor controlled with a single axis programmable controller and microstep driver that interfaces with in house LabVIEW 2011 software (National Instruments Corporation, Austin, TX). The tank was positioned such that there was no air gap between it and the radiosurgery cone. Physical limitations of our experimental setup limited the minimum depth of measurement along the beam central axis to 40 mm for the Markus chamber and 42 mm for the PR60020 diode. The diode detector was also used to measure transverse dose profiles with the beam incidence perpendicular to the axial direction of the detector (i.e., the detector was in edge‐on orientation) to increase spatial resolution (Fig. 3).

### B. Film metrology

9×5 cm pieces of Gafchromic EBT2 film (Advanced Materials Group, Wayne, NJ) were placed in custom designed holders and positioned in a water tank at WEDs corresponding with diode cross‐profile data, as shown in Table 1. Using the same energies and treatment nozzle configurations as analogous diode data (see Table 1 and above), films were irradiated for 165 s (corresponding to a dose range of approximately 3.5 to 7.3 Gy depending on energy, modulation, and water‐equivalent depth). Films were scanned in 48 bit RGB format at 72 dpi using an Epson 10000XL flatbed scanner. Dose maps where created with Film QA Pro software (Ashland Inc., Wayne, NJ) using a triple‐channel dosimetry method based on a rational function‐fit to previously exposed calibration films.^20^, ^21^ Cross‐sectional profiles of the dose maps were obtained using Film QA Pro and the data was normalized based on the maximum dose value. Film cross‐profiles were center‐aligned with diode cross‐profiles for analysis.

### C. Monte Carlo simulations

**Figure 4:**
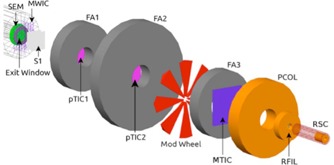
Schematic diagram of the LLUMC Gantry 1 treatment nozzle with radiosurgery cone geometry used in Monte Carlo simulations. From left to right the components are: the secondary emission monitor (SEM) and titanium exit window, multiwire ion chamber (MWIC), initial scatterer (two lead wedges) (S1), first proton transmission ionization chamber (pTIC1), first steel fixed aperture (FA1), second pTIC2, second fixed aperture (FA2), Perspex modulation wheel, third fixed aperture (FA3), multielement transmission ion chamber (MTIC), brass precollimator (PCOL), ridge filter (RFIL), and radiosurgery cone (RSC).

Computer simulations were performed using software developed in‐house that incorporates the Geant4.9.6 Monte Carlo particle simulation toolkit.^22^, ^23^ Protons and secondary particles were tracked through a model of our Gantry 1 treatment nozzle using a custom physics list incorporating binary cascade models of inelastic interactions of hadrons and heavy ions, low‐energy Livermore physics models for electromagnetic interactions, and the high‐precision neutron package^(24)^ similar to that used by Wroe et al.^(25)^ and McAuley et al.^(26)^ (see Fig. 4). The latter two model sets allow more accurate tracking of dose deposited by secondary particles. Proton energies and treatment nozzle parameters were chosen to correspond to the diode, ion chamber, and film experiments described above and which are summarized in Table 1.

Simulated 127 or 157 MeV protons were delivered through a single scattering system to a voxelized water phantom. Phantom voxel size was 0.5×0.5×0.5 mm (with range cuts of 250 μm) and $32\times 10^{8}$ histories were used without bias for each energy/modulation combination. Total dose deposited from primary and secondary particles was scored in each voxel and longitudinal depth dose (average using four central voxels), and transverse cross‐sectional dose (individual voxels) profiles were calculated from the phantom data using software developed in Python.^(27)^ Modulated depth profiles were normalized with respect to dose at the center of modulation (COM) of the spread‐out Bragg peak. Unmodulated depth‐dose profiles and transverse profiles were normalized by maximum dose. Simulation transverse profiles were center‐aligned with diode cross‐profiles for analysis.

To assess LET dependence in the diode response, the ratio of relative diode dose to relative Markus dose for the modulated beams was plotted against dose‐weighted average lineal energy that was calculated from Monte Carlo simulations. Because lineal energy is the stochastic analog of LET, lineal energy independence is sufficient to imply LET independence. In addition, unlike LET, it is readily calculated for single‐event depositions involving both primary and secondary particles at microscopic scales. These simulations used voxels that were 0.02 mm thick along the beam axis and 0.05×0.05 transverse to the axis (range cuts were 10 μm). Single‐event energy depositions involving primary and secondary particles (e.g., electrons, neutrons, gammas, alphas) were scored in a set of voxels that formed transverse slices, centered on the beam axis, that were one voxel deep and 20×20 wide. Lineal energy was determined for each voxel in the slice by dividing the energy deposited in the voxel by the average chord length. Because of the small size and proximity of the voxels to the beam axis, this length was taken as 0.02 mm (i.e., the voxel thickness along the beam axis). Thus, lineal energy was sampled over 400 voxels that collectively had the same thickness and cross‐sectional area as the sensitive volume of the diode detector. The dose‐weighted average lineal energy $y_{D}$ (Eq. (1)) was then calculated as the ratio of the second and first moments of lineal energy for each slice along the respective depth‐dose curve, according to Eqs. (1) and (2):^(28)^

(1) $$y_{D}=\frac{1}{y_{F}}\int_{0}^{\infty }y^{2}f_{1}(y)dy$$

(2) $$y_{F}=\int_{0}^{\infty }yf_{1}(y)dy$$

where *y* is lineal energy, $f_{1}(y)$ is the single‐event probability distribution, and $y_{F}$ is the frequency‐weighted average linear energy.^(28)^

## III. RESULTS

**Figure 5:**
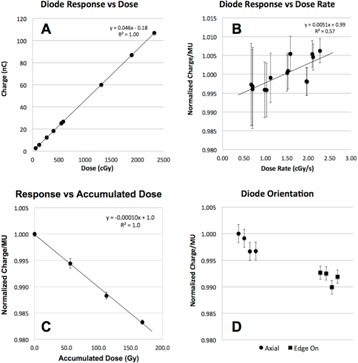
Plot (a) showing that diode response is linear with respect to dose from 0.6 to 23 Gy. Plot (b) showing diode response is uniform with respect to dose rate from 0.7 to 2.3 cGy/s. The diode (c) displays a linear decrease in response of ∼1% per 100 Gy. Plot (d) showing diode response is essentially independent of orientation (i.e., whether detector is oriented such that the beam is incident parallel or perpendicular to the detector axis).

Results of the precursory experiments are shown in Fig. 5. The high correlation and small intercept (∼100 pC) of Fig. 5(a) reveals the diode response was linear with respect to dose over a dose range from 0.6 to 23 Gy. Figure 5(b) shows the response was also uniform with dose rate over a range of 0.7 to 2.3 cGy/s. In addition, the diode displayed a linear decrease in response with accumulated dose of ∼1% per 100 Gy. Finally, Fig. 5(d) shows that the diode response in the axial and edge‐on orientations agreed within 0.6%. The error bars in Fig. 5 were calculated using standard error propagation in (a), (b), and (c), and standard deviation (SD) for each set of orientation measurements in Fig. 5(d). When not shown, error bars are smaller than symbol size.

**Figure 6:**
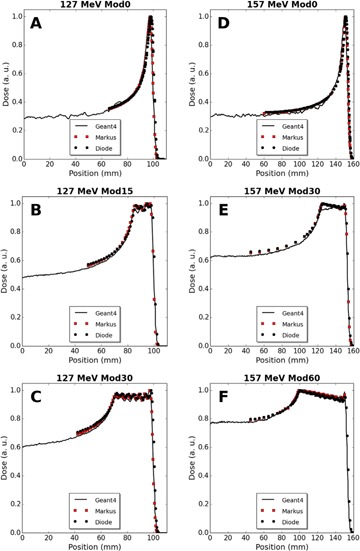
Depth‐dose comparisons of diode, Markus ion chamber, and Geant4 simulation data for 127 MeV protons: (a) unmodulated (Mod0), (b) 15 mm modulation (Mod15), (c) 30 mm modulation (Mod30); and for 157 MeV protons: (d) (Mod0), (e) 30 mm modulation (Mod30), and (f) 60 mm modulation (Mod60).

The depth‐dose profiles shown in Fig. 6 display good qualitative agreement between ion chamber, diode, and simulation data across all modulations and both energies. Table 2 provides a quantitative analysis of depth‐dose profile data between the diode, ion chamber, and simulation and lists the water‐equivalent depth of 50% maximum distal dose (D50) for the various data modalities. For diode versus the Markus ion chamber, D50 agreement was less than 0.9 mm for all measurement cases (corresponding to a discrepancy of less than 0.9% of the diode D50).

The maximum diode versus simulation D50 discrepancy for all beams was 1.1 mm, and all discrepancies were less than 1.1% with respect to diode D50.

**Figure 7:**
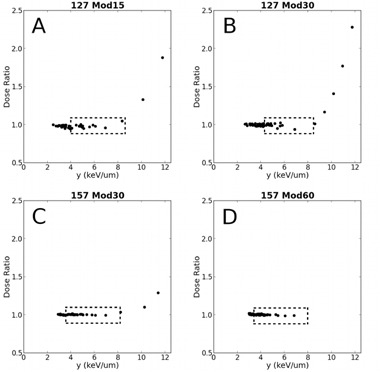
Plot of the relative dose ratio of the detectors vs. dose‐weighted lineal energy (y) calculated from Monte Carlo simulations for 127 MeV protons: (a) 15 mm modulation (Mod15), (b) 30 mm modulation (Mod30); and for 157 MeV protons: (c) 30 mm modulation (Mod30), (d) 60 mm modulation (Mod60). The dashed rectangles represent the spread‐out Bragg peak (90% to 90% dose region) for simulated depth‐dose curves and ratios are plotted up to the 25% distal dose (full data not available for 157 MeV Mod60).

Figure 7 displays graphs of the ratio of relative dose measured by the diode detector to relative dose measured by the Markus chamber plotted against the simulated dose‐weighted average lineal energy deposited by 127 and 157 MeV modulated beams as a function of depth. The dashed rectangles correspond to the spread‐out Bragg peak (90% to 90% dose region) (SOBP) of the simulated depth‐dose curves and ratios are plotted up to the 25% distal dose. A sharp rise in ratio beginning near the distal edge of the SOBP is apparent. Second order interpolated ratios at the distal edges of the SOBP (i.e., the 90% distal dose) are 1.07, 1.0, and 1.03 for 127 MeV Mod15, 127 MeV Mod30, and 157 MeV Mod30, respectively (full ratio data are not available for 157 MeV Mod60). The respective ratios at the 95% distal dose — which in each case corresponds to a depth less than 0.5 mm shallower than the 90% values — are 1.01, 0.96, and 1.01.

**Table 2:** Water‐equivalent depth of 50% maximum distal dose

*Energy (MeV)*	*Modulation (mm)*	*Diode (mm)*	*Markus (mm)*	*Simulation (mm)*	*Diode – Markus (mm)*	*Diode – Geant4 (mm)*
127	None	100.8	99.9	99.5	0.9	1.2
127	15	100.4	99.5	99.8	0.9	0.7
127	30	100.5	99.6	99.8	0.9	0.7
157	None	154.2	153.7	153.1	0.5	1.2
157	30	153.6	153.2	153.0	0.4	0.6
157	60	153.8	–^a^	153.0	–^a^	0.8

a Data unavailable.

**Figure 8:**
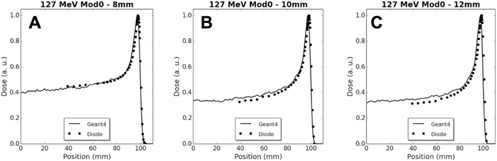
Depth‐dose plots showing diode and simulation data for unmodulated 127 MeV beams with initial diameters of (a) 8 mm, (b) 10 mm, and (c) 12 mm.

**Figure 9:**
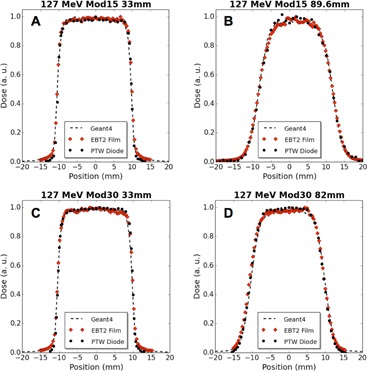
Transverse dose profile comparisons of diode, film, and Geant4 simulation data for modulated 127 MeV proton beams at 33 mm WED and COM: (a) 15 mm modulation (Mod15) at 33 mm, (b) Mod15 at 89.6 mm, (c) 30 mm modulation (Mod30) at 33 mm, and (d) Mod30 at 82 mm.

**Figure 10:**
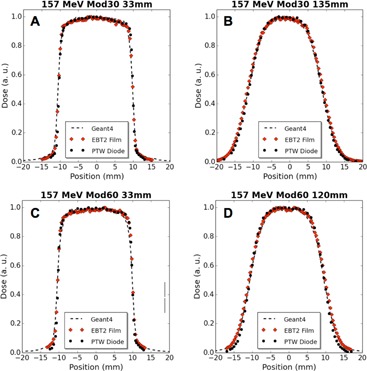
Transverse dose profile comparisons of diode, film, and Geant4 simulation data for modulated 157 MeV proton beams at 33 mm WED and COM: (a) 30 mm modulation (Mod30) at 33 mm, (b) Mod30 at 135 mm, (c) 60 mm modulation (Mod60) at 33 mm, and (d) Mod60 at 120 mm.

Figure 8 shows depth dose plots for 127 MeV unmodulated 8, 10, and 12 mm beams compared with analogous Monte Carlo simulations. Figures 9 and 10 provide measured and simulated transverse dose profile data for both energies and various beam modulations and depths. The PTW proton diode was compared experimentally to Gafchromic EBT2 film and also to Geant4‐based simulations. Qualitatively, these figures display good agreement between the three modalities, with no significant differences in beam profile. In an edge‐on orientation (i.e., with the beam axis perpendicular to the detector central axis), the PTW proton diode exhibited spatial resolution that was comparable with typical applications of film. This is demonstrated in Fig. 9 and 10 where, along the beam penumbra (between 20% and 80% maximum dose), the diode dose matches well with film and simulation where the diode data were acquired with 0.25 mm spacing (Fig. 9(a) and (c), Fig. 10(a) and (c)), and 0.5 mm spacing (Fig. 9(d), Fig. 10(b) and (d)).

Tables 3 and 4 provide a quantitative comparison in beam profile data for the PTW diode, EBT2 film, and Geant4‐based simulations. Experimentally, the PTW diode and EBT2 film reported FWHM and FW90M within 0.7 mm with an average difference of 0.3 mm (corresponding to an average discrepancy of 1.6% with respect to diode widths). When comparing the PTW diode to simulation results, the difference in FWHM has a maximum discrepancy of 0.3 mm and an average discrepancy of 0.1 mm (average discrepancies of 0.5% with respect to diode values). At the FW90M, level the differences were larger, with a maximum discrepancy of 1.5 mm and an average discrepancy of 0.9 mm (corresponding to a 6% average discrepancy with respect to diode).

**Table 3:** Full‐width parameters of dose cross‐profiles from diode, film, and simulation data for 127 MeV beams

*Energy (MeV)*	*WED (mm)*	*Modulation (mm)*		*Diode (mm)*	*Film (mm)*	*Simulation (mm)*	*Diode – Film (mm)*	*Diode – Geant4 (mm)*
127	33	15	FW90M	18.0	18.3	17.6	−0.3	0.4
127	33	15	FWHM	20.1	20.4	20.2	−0.2	−0.1
127	89.6	15	FW90M	12.2	12.7	13.4	−0.5	−1.2
127	89.6	15	FWHM	19.7	20.0	20.0	−0.4	−0.3
127	33	30	FW90M	18.0	18.0	17.4	0.0	0.6
127	33	30	FWHM	20.2	20.3	20.2	−0.2	0.0
127	82	30	FW90M	14.8	14.9	14.3	−0.1	0.5
127	82	30	FWHM	20.1	20.4	20.1	−0.3	0.1

FWHM = full width at half maximum dose; FW90M = full width at 90% maximum dose.

**Table 4:** Full‐width parameters of dose cross‐profiles from diode, film, and simulation data for 157 MeV beams

*Energy (MeV)*	*WED (mm)*	*Modulation (mm)*		*Diode (mm)*	*Film (mm)*	*Simulation (mm)*	*Diode – Film (mm)*	*Diode – Geant4 (mm)*
157	33	30	FW90M	18.4	18.2	17.6	0.2	0.8
157	33	30	FWHM	20.2	20.3	20.3	−0.1	−0.1
157	135	30	FW90M	12.3	12.1	11.4	0.2	0.9
157	135	30	FWHM	20.1	20.7	20.1	0.6	−0.1
157	33	60	FW90M	18.1	18.3	17.0	−0.2	1.1
157	33	60	FWHM	20.2	20.3	20.3	−0.1	−0.1
157	120	60	FW90M	13.9	13.4	12.4	0.4	1.5
157	120	60	FWHM	20.3	20.9	20.2	−0.6	0.1

FWHM = full width at half maximum dose; FW90M = full width at 90% maximum dose.

## IV. DISCUSSION

### A. Linearity, dose rate, orientation, and radiation damage

Figure 5 shows that the diode response was linear with respect to dose and uniform with dose rate over a range of doses and dose rates relevant to radiosurgery. The detector could be orientated either parallel or perpendicular to the beam for depth‐dose or beam‐profile measurements, respectively, with a differential response of less than 1%. In addition, the diode exhibited a linear decrease in response with accumulated dose of ∼1% per 100 Gy. This loss of sensitivity is a well‐known result of radiation damage typical in silicon diodes^(29)^ where Si nuclei are displaced by particle collisions.^(11)^ Since such displacements occur more often in proton irradiation, loss of sensitivity is greater than is seen with photon radiation.^(11)^ Because sensitivity tends to decrease following initial radiation exposure, the detector, as is common, comes preirradiated from the manufacture to minimize effect. While the presence of sensitivity loss requires periodic recalibration for accurate absolute dosimetry, due to the low magnitude of the effect, it can be neglected over the course typical QA operations and does not affect relative dosimetric measurements.

### B. LET dependence

Depth‐dose profiles comparing diode and ionization data were in good agreement over the full particle range (see Fig. 6). In particular, it is important to note that no significant discrepancies were evident over the SOBPs of the curves. The SOBP is composed of a superposition of pristine Bragg peaks that represent a spectrum of particle energies which is further spread by proton straggling that increases as a function of particle energy. The lack of deviation of the diode data compared to Markus and simulation data in the SOBP region, and especially near the end of the SOBP where straggling and mean energy is the greatest, suggests that effects of LET are negligible for the energies considered in the present work.

Plots of diode to Markus dose ratio versus simulated average lineal energy further highlighted a negligible LET‐dependent diode response across the SOBP to the distal 95% dose level. The sharp rise observed in the data of Fig. 7 beyond the 95% distal dose can be attributed to either a slight difference in relative alignment in the steep distal region of the two depth‐dose curves (Table 2), an overresponse of the diode at and beyond the distal portion of the SOBP, or a combination of the two factors. As evidence for the former case, Table 2 shows that the difference between the diode and Markus D50 values are 0.4 to 0.9 mm. If we assume this (positive) difference is purely due to misalignment, then at a given depth along the distal edge of the depth‐dose curve the Markus doses would be represented as lower than they actually are. Assuming a linear falloff of dose from 95% to 25%, shifts of 0.4 and 0.9 mm are expected to result in a change in ratio on the order of 10% to 30%. A careful examination of Fig. 6 shows that points along the distal edge at corresponding depths have dose differences consistent with theses shifts (8% to 30%), supporting the conclusion that the rise in ratio beginning in the very distal portion of the SOBP is due to differences in alignment between detectors. However, as the largest fraction of stopping protons occurs at the SOBP distal edge, resulting in a higher LET, it cannot be ruled out that the detector would have a nonuniform response in this region. In any case, while further investigation into the response of the diode in the high‐LET distal SOBP and edge region is warranted and, in the interim, caution should be exhibited when interpreting measurements in this region, we can safely conclude that, for the clinically relevant radiosurgery beams considered in our study, the diode detector exhibited a negligible LET dependence from entrance up to the very distal portion of the SOBP.

### C. High spatial resolution

The small axial cross‐sectional area of the diode SV (1 mm^2^) allows for measurements in small fields without partial volume averaging that can occur with larger dosimeters. The measured D50 between the diode and PTW Markus N23343 plane‐parallel ion chamber was within 1% of the diode D50 for all energies and beam modulations (Table 2). In addition, D50 values of simulated data matched diode data to within 1.1%. This, taken together with the good qualitative agreement of the diode, Markus and Monte Carlo depth dose curves of Fig. 6 demonstrate the utility of the Monte Carlo simulations to predict dose deposited and measured by the diode detector in this energy range. Thus, the Monte Carlo simulations of Fig. 8 suggest that accurate dose measurements of smaller diameter beams (i.e., 8, 10, 12 mm) are achievable with the PR60020 detector. Furthermore, the 20 μm thickness of the SV rivals the 28 μm nominal thickness of the active layer in EBT2 and EBT3 Gafchromic film^(21)^ and allows high‐density point measurements parallel and perpendicular to the beam axis.

The small SV of the diode also reduces detector size effects on transverse beam profiles that become more important as field size decreases. The reduction becomes more pronounced when profile data are collected in edge‐on orientation. Assuming a linear relationship with slope = 1 between detector size‐induced deviations in penumbra width and axial detector radius, r,^30^, ^31^ deviations over the profile penumbra are expected to be ∼0.5r or 0.3 mm for the diode detector in axial orientation. In edge‐on orientation, the effective detector cross‐section becomes rectangular in shape and over an order of magnitude smaller in width (i.e., 20 μm). Equations 11 and 12 from the study by Sahoo et al.^(31)^ can be used to show the ratio of edge‐on to axial deviations is 0.03. Thus, the detector size effect is expected to be ∼0.009 mm and unmeasurable. Evidence for a measurable effect could manifest itself as positive differences between diode and simulation FW90M widths; however, Tables 3 and 4 show that seven of eight of these differences are negative. Although a rigorous assessment of detector size effect is beyond the scope of the present work and knowledge of size effects in axial orientation is important, the data presented for edge‐on orientation do not appear to show any such effects.

### D. Cross‐profile measurements

When arranged in edge‐on orientation with the 20 μm thick SV perpendicular to the beam central axis, beam profile measurements were possible with a high degree of spatial resolution comparable with typical applications of film (e.g., scanned at 72 dpi (0.35 mm spacing)). This submillimeter spatial resolution is of great advantage for the small fields employed in proton radiosurgery and regions of high‐dose gradient. Agreement between the diode and EBT2 film FWHM and FW90M was within 0.7 mm for all depths, beam energies, and modulations. The main advantages of using radiochromic film as a dosimeter include submillimeter spatial resolution and the ability to capture continuous 2D dose distributions. However, several technical and ease‐of–use limitations must be controlled and accounted for when using film.^1^, ^21^, ^32^, ^33^ Recent improvements in technology and techniques such as multiple‐channel dosimetry,^(20)^ simplified calibration and intralot recalibration,^(21)^ calibration‐less relative dosimetry,^(34)^ and film that is less sensitive to light, have ameliorated concerns about artifact effects and ease of use, but have not eliminated them. In contrast to film, diode detectors allow near real‐time dose readout without processing delays or supporting data acquisitions, and do not require special storage and handling. The diode detector tested here provided a useful tool for 1D profiles, but did require additional setup time over film and could not produce 2D dose distributions. However, as diode technology improves, arrays of small closely packed diode SVs^35^, ^36^, ^37^ could be used to produce 2D dose maps in real‐time without the difficulties associated with film.

Finally, although it provides significant spatial resolution benefits when measuring lateral beam profiles, it is important to note the edge on orientation is not indicated by the diode manufacturer. In addition, while the manufacturer specifies a WET of the casing in the axial dimension, no such specification is given for the perpendicular orientation. However, our results show that, by simply centering the SV with respect to depth in each orientation, accurate results are possible. In any case, good practice suggests that each device should be tested before assuming an equivalent response in each orientation.

## V. CONCLUSIONS

The goal of this work was to evaluate the PTW PR60020 diode in proton fields typically associated with radiosurgical applications at LLUMC. Its rugged and water‐proof construction allows for easy deployment in water tanks, solid water, and phantoms that are commonly used in proton radiosurgery QA, without special handling considerations. Dosimetrically, the diode displayed a linear response with dose and dose rate, a small sensitivity decrease per unit dose (0.01%/ Gy), and a small axial/edge‐on differential response (<1%). In addition, the diode exhibited negligible LET dependence from entrance to the very distal portion of the SOBP for particle energies and ranges typical for clinical radiosurgery. The small sensitive volume (cross‐sectional area of 1 mm^2^ and 20 μm thickness) allows for high spatial resolution measurements in areas of large‐dose gradients (e.g., penumbra) and small treatment fields, while maintaining sufficient sensitive volume to ensure measurements could be completed without excessive beam delivery. In edge‐on orientation, it facilitated the acquisition of beam profiles with a spatial resolution comparable to both Monte Carlo simulations and film measurements. It is expected that this detector will be deployed for clinical use in proton radiosurgery and other small field proton applications, including active beam scanning dosimetry at our facility.

## ACKNOWLEDGMENTS

The team would like to acknowledge the support of the accelerator operations team at LLUMC for their support in beam delivery, water tank development, and operation. This project was sponsored in part by funding from the Department of Defense (DOD W81XWH‐BAA‐10‐1).

## Supporting information

Supplementary Material

Supplementary Material
