# Supplementary figures and images for: Evaluation of the dosimetric properties of a diode detector for small field proton radiosurgery
Source: J Appl Clin Med Phys. 2015 Nov 8;16(6):51–64. doi: 10.1120/jacmp.v16i6.5391 (PMC5691016; doi:10.1120/jacmp.v16i6.5391)

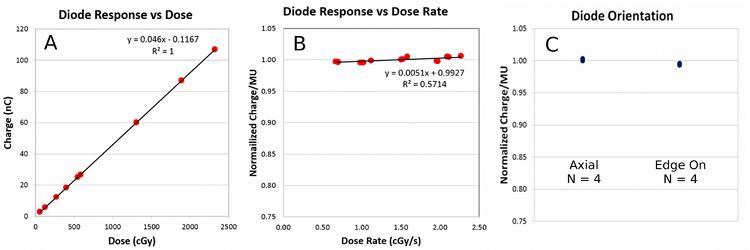

Supplement: Supplementary file 1 — Supplementary Material [file ACM2-16-051-s001.png]

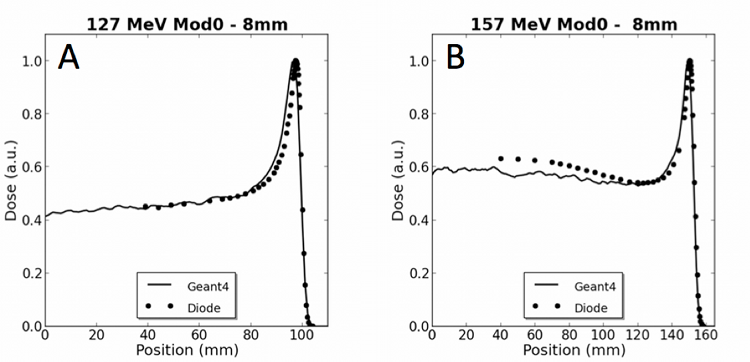

Supplement: Supplementary file 2 — Supplementary Material [file ACM2-16-051-s002.png]
